# An Industrial Internet Security Assessment Model Based on a Selectable Confidence Rule Base

**DOI:** 10.3390/s24237577

**Published:** 2024-11-27

**Authors:** Qingqing Yang, Shiming Li, Yuhe Wang, Guoxing Li, Yanbin Yuan

**Affiliations:** College of Computer Science and Information Engineering, Harbin Normal University, Harbin 150025, China; yangqq@stu.hrbnu.edu.cn (Q.Y.);

**Keywords:** rule based belief system, expert system, industrial internet, choosing covariance matrix adaptive evolution strategy, selective modeling criteria

## Abstract

To mitigate the impact of network security on the production environment in the industrial internet, this paper proposes a confidence rule-based security assessment model for the industrial internet that uses selective modeling. First, a definition of selective modeling tailored to the characteristics of the industrial internet is provided. Based on this, the assessment process of the Selectable Belief Rule Base (BRB-s) model is introduced. Then, in combination with the Selection covariance matrix adaptive evolution strategy (S-CMA-ES) algorithm, a parameter optimization method for the BRB-s model is designed, which expands the selective constraints on expert knowledge. This model establishes a better unidirectional selection strategy among different subgroups, and while expanding the selection constraints on expert knowledge, it achieves better evaluation results. This effectively addresses the issue of reduced modeling accuracy caused by insufficient data and poor data quality. Finally, the experiments of different evaluation models on industrial data sets are compared, and good results are obtained, which verify the evaluation accuracy of the industrial Internet network security situation assessment model proposed in this paper and the feasibility and effectiveness of the S-CMA-ES optimization algorithm.

## 1. Introduction

With the popularization of the internet and the application of intelligent technology, the interconnection and complexity of the industrial internet are increasing, the number of data flows and devices is expanding, and the risk of network attacks is also increasing [1]. The industrial internet is a key component of the industrial production process, and its network security directly affects the normal operation of industrial production.

The industrial control system, as the core part of the industrial internet, will cause immeasurable economic losses or serious negative impacts once attacked and damaged [2]. For example, in February 2023, the Irish Data Protection Commissioner (DPC) imposed a maximum fine of EUR 405 million on Instagram for Meta’s alleged failure to protect children’s personal data. In March of the same year, the French data protection regulatory agency imposed a fine of EUR 5 million (approximately USD 5.4 million) on the short video platform TikTok for violating cookie consent rules. In April of the same year, Seagate, a major manufacturer of mechanical hard drives, agreed to pay a fine of USD 300 million and reached a settlement with US authorities over violating US export control laws by shipping hard drives worth over USD 1.1 billion to Huawei in China. In November of the same year, Huamei Bank Limited was ordered to rectify its actions and was fined RMB 600,000 for “insufficient production environment safety control” and “insufficient production data safety control”. Therefore, establishing an accurate and reliable network security assessment model is particularly important [3].

To avoid losses caused by attacks as much as possible, it is particularly important to conduct security assessment and risk identification on the security of the industrial internet in a timely manner [4], and use the evaluation results to reflect the security and health status of the industrial internet in a timely manner, which can provide network administrators with safe, reliable, and efficient security measures and suggestions [5].

To address the issue of risk assessment on the industrial internet, common approaches involve improving the accuracy of risk assessment through various modeling methods. Currently, the main technologies used to build assessment models include blockchain technology [6], feature selection techniques [7], intrusion detection technology [8], digital twin technology [9], and others. However, these commonly used evaluation models still have significant limitations: they are heavily reliant on the quality and quantity of data.

1. Blockchain-based technology: Using blockchain technology for assessment modeling offers features such as decentralization, data tamper-resistance, transparency, and high security. Through the generalized additive blockchain modeling technique, an end-to-end mechanism can be implemented to increase data authenticity [10]. It can also improve the efficiency and security of supply chain financial systems by enhancing information sharing and transparency, reducing the risk of financing defaults, and automating smart contracts [11]. Furthermore, it can integrate blockchain-based data models to ensure data transparency and achieve decentralized blockchain integration with SDN [12,13]. However, blockchain technology’s reliance on the integrity and completeness of historical data for constructing risk assessments means that the model’s accuracy may decline when data are insufficient or missing.
2. Based on feature selection technology: Using feature selection techniques for assessment modeling allows for the extraction of key features from large amounts of sensor and operational data, supporting data-driven decision-making. By filtering and quantifying the uncertainties in the industrial internet environment, more effective models can be developed. A similar approach is also applicable to the Maximum Relevance Minimum Redundancy (mRMR) algorithm [14]. A new feature is the selection of algorithms for image quality assessment without reference [15], unsupervised multi-view feature selection [16], and feature level fusion algorithms [17]. However, feature selection-based models require extracting key features from large amounts of data and optimizing production processes through data-driven approaches. If the data quality is poor or the data volume is insufficient, it will result in a decline in the accuracy and reliability of the assessment outcomes.
3. Intrusion detection technology: Through real-time monitoring of equipment and network activities in the industrial internet environment, using intrusion detection technology for modeling allows for the rapid identification of security threats and abnormal behaviors, providing strong assessment capabilities for the industrial internet environment. An evaluation model can be established through quantitative strength evaluation of industrial internet attacks [18] or combined with an intrusion detection evaluation model based on deep extreme value learning machine [19] or an intrusion detection industrial internet evaluation model based on deep learning [20]. However, intrusion detection technology heavily relies on the real-time nature and accuracy of data. Incomplete or inaccurate data may lead to false positives or missed detections.
4. Digital twin modeling: Digital twin modeling enables the creation of virtual models of physical systems, allowing for real-time monitoring of equipment status and conducting safety tests without impacting actual operations. Common modeling techniques for digital twins include evaluative modeling based on digital twins [21,22], virtualization and simulation modeling based on digital twins [23], and data-driven modeling [24], which is based on deconvolution digital twin modeling [25]. Modeling is based on behavioral models [26], and digital twins are based on marginalization [27]. However, digital twin modeling is even more dependent on data, especially in real-time monitoring and prediction processes. Incomplete or poor-quality data can significantly impact the accuracy of the model.

The four traditional modeling methods mentioned above are somewhat inadequate when faced with small sample sizes and poor-quality data. To address the issue of reduced modeling accuracy due to insufficient data, the BRB-s model was designed. The BRB-s model can effectively utilize engineering experience and expert knowledge to fuse small sample monitoring data with strong, nonlinear modeling capabilities and high modeling accuracy. To further improve the accuracy of the model, a modeling strategy that uses different subcategory groups to make unilateral decisions for the superior strategy is adopted. The BRB-s model further expands the selective constraints on expert knowledge by expanding them, combining it with the S-CMA-ES algorithm, and establishing a better one-way choice between different subcategory groups; thus, the selective constraints on expert knowledge are further expanded. A series of industrial internet network security assessment models are constructed based on the data of evidential reasoning (ER) iterative fusion and actual data. Compared with the ordinary belief rule base (BRB) model, the BEB-s model improves the accuracy of the assessment model by optimizing the one-way selection path and the selective constraints on expert knowledge.

The first part of this paper describes the shortcomings of traditional modeling methods, and proposes an industrial internet assessment model based on BRB-s on the basis of revealing the original modeling. The second part provides a preliminary overview of the security situation assessment within the industrial internet network. The third section constructs the BRB-s industrial internet assessment model by combining it with the S-CMA-ES algorithm. In the fourth section, through specific experiments, the BRB-S model is compared with other modeling methods employing different strategies, yielding more favorable comparative results. This validates that the BRB-s model outperforms other evaluation models in network security assessment, particularly in industrial internet environments characterized by insufficient data and poor data quality. A feasible solution is proposed to address the common challenges of insufficient data and poor data quality in industrial internet environments. Finally, the fifth part provides a summary.

## 2. Problem Description

The security situation assessment of the industrial internet includes two parts: assessment index fusion and security situation assessment. Considering the complexity of the industrial internet environment, this experiment integrates multiple indicators that affect the network security of the industrial internet. By using the ER iterative algorithm, multiple indicators are progressively merged using a hierarchical fusion strategy, merging multiple layers of uncertain indicators.

Through the application of the BRB-s model, combined with the S-CMA-ES algorithm, the parameters of the evaluation process were optimized, the selectivity of expert knowledge was expanded, and an industrial internet network security evaluation model was developed.

To facilitate the integration of evaluation indicators, the four levels of evaluation indicators of the industrial internet are defined as $A_{xyz},B_{xy},C_{x}$, and *D*.

The first step is to evaluate the following set of indicators:

(1) $$\begin{array}{c} {AA}_{xyz}=\left\{\left.{aa}_{xyzk}\right|x=1,2;y=1,2,3;z=1,2,3,4,5;k=1,2\right\} \end{array}$$

(2) $${BB}_{xy}=\left\{\left.{bb}_{xyz}\right|x=1,2;y=1,2,3;z=1,2,3,4,5\right\}$$

(3) $${CC}_{x}=\left\{\left.{cc}_{xy}\right|x=1,2;y=1,2,3\right\}$$

(4) $$\begin{array}{c} DD=\left\{\left.{dd}_{x}\right|x=1,2\right\} \end{array}$$

In Equations (1)–(4), ${AA}_{xyz}$, ${BB}_{xy}$, ${CC}_{x}$ and *DD* are used as the fourth, third, second, and first evaluation indicator sets, respectively. Among them, ${aa}_{xyzk}$, ${bb}_{xyz}$, ${cc}_{xy}$, and $dd_{x}$ are the fourth, third, second, and first evaluation indicators, respectively.

Then, on the basis of Equations (1)–(4), the corresponding fusion indicators are obtained:

(5) $${bb}_{xyz}=ER\left({AA}_{xyz},\gamma \right)$$

(6) $${cc}_{xy}=ER\left({BB}_{xy},\beta \right)$$

(7) $${dd}_{x}=ER\left({CC}_{x},\alpha \right)$$

*ER* (•) represents the fusion nonlinear transformation process based on the *ER* iterative algorithm evaluation index, and *α*, *β*, and *γ* represent the parameter set of *ER*. ${bb}_{xyz}$ represents the third level evaluation indicator obtained by fusing all evaluation indicators in the fourth level ${AA}_{xyz}$, ${cc}_{xy}$ represents the second level indicator obtained by fusing all evaluation indicators in the third level ${BB}_{x,y}$, and ${dd}_{x}$ represents the first level indicator obtained by fusing all evaluation indicators in the second level ${CC}_{x}$.

Obtain the situation assessment result y on the basis of the fusion result:

(8) $$\begin{array}{c} y=BRB\left(a_{1},a_{2},\eta \right) \end{array}$$

*BRB* (•) represents nonlinear transformation, which is based on the process of *BRB*. $a_{1}$ and $a_{2}$ represent the input parameters of the *BRB* model, while η represents the *BRB* parameter set.

## 3. The BRB-s Evaluation Model Based on the Integration of S-CMA-ES and ER

### 3.1. Assessment Process

**Figure 1:**
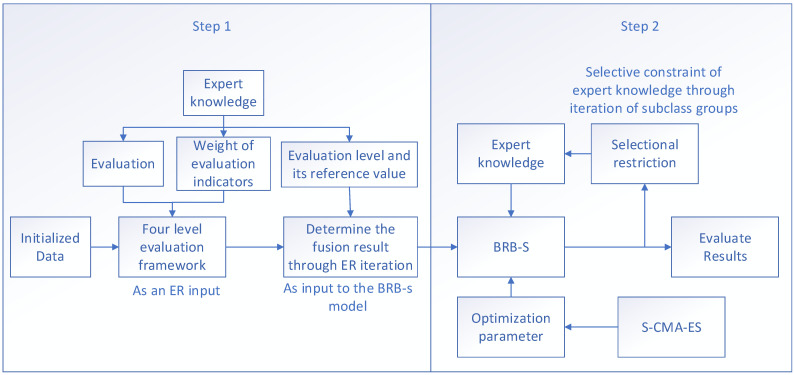
Model evaluation framework.

The evaluation process of the situation assessment model established in this paper consists of two main parts, as shown in Figure 1.

Step 1: First, decision-makers establish appropriate evaluation criteria on the basis of factors affecting the network security assessment of the industrial internet. Suitable reference values are then set through these evaluation criteria. The different indicators are subjectively stratified, and the uncertainties between the indicators are gradually integrated via the ER approach to obtain the final integrated result for each indicator.

Step 2: The integrated results are used as input to the BRB-s model. The S-CMA-ES algorithm is employed to divide the results into multiple subset groups, which apply selective constraints on expert knowledge. This reduces the impact of uncertainty in expert knowledge and enhances the evaluation accuracy of the model.

### 3.2. Evaluation Criteria and Their Functions

#### 3.2.1. Evaluation Criteria

**Figure 2:**
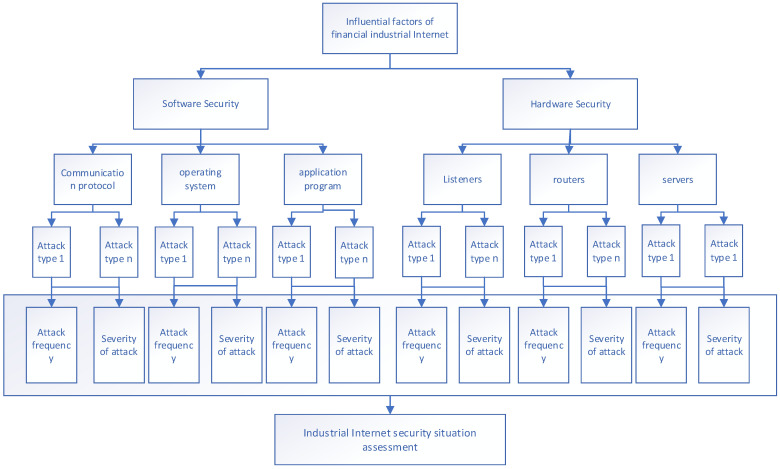
Four-level evaluation framework using ER fusion.

Owing to the complexity of the industrial internet environment, this paper focuses solely on external attacks encountered by the industrial internet. To address the external variables affecting the network security of the industrial internet and to align with real-world environments and collected data, Figure 2 presents a four-level evaluation framework of the ER fusion process.

The external attacks faced by the industrial internet can be broadly categorized into hardware-related attacks and software-related attacks. On the basis of this classification, and considering the hierarchical structure of software, attacks targeting applications, operating systems, and communication protocols are established as secondary evaluation criteria. On the hardware side, servers, routers, and monitors are designated as secondary evaluation criteria on the basis of their functionalities. These modules are primarily vulnerable to various attacks, including Denial of Service Attack (DOS), Distributed Denial of Service Attack (DdoS), ransomware attacks, scanning attacks, backdoor attacks, injection attacks, and others. The methods of attack vary for different devices, resulting in different impacts. Among these attacks, only four are likely to affect software or hardware devices. Therefore, these potentially threatening attack methods are treated as secondary evaluation criteria corresponding to their respective attack points. The impact of these attack methods on different components also varies, as does their frequency. The attack frequency is determined by the number of attacks experienced over a certain period. By combining expert knowledge and the results of actual investigations, the severity of the attack on each module is assessed. By integrating the determined attack frequency with the severity of the attack, a four-level evaluation framework for the industrial internet is constructed.

After the establishment of industrial internet evaluation indicators, decision-makers determine the proportion of each indicator (ω) through expert knowledge. Moreover, to comprehensively establish an industrial internet evaluation framework, experts collect only the required indicators and establish a four-level evaluation framework, as shown in Table 1, which presents the distribution weight of each evaluation indicator (r) (ω).

Considering the volatility of indicators in the security assessment of the industrial internet, the difference between them is not significant, so a method sensitive to slight differences is needed. This idea conforms to the principle of the coefficient of variation method [28], which assigns weights to different indicators through the use of the variance method strategy. The calculation process is summarized as follows.

The core logic of the four-layer evaluation framework is to sequentially extract, integrate, and weight the information from different layers, ultimately obtaining the two input premises required by the model. Starting from the fourth layer, the weights between the objects to be fused at each layer are determined using the variation method. The integrated indicators of each layer, representing the type of attack suffered, are then used to generate the next layer’s indicators. This process continues until the two input premises required by the model are obtained.

Step 1: Derive the initial indicator data matrix using the fourth level evaluation indicators

(9) $$X={\left(X_{i,\dot{j}}\right)}_{n\times m}\left(i=1,\ldots ,n;j=1,\ldots ,m\right)$$

where $x_{i,j}$ is the *j*th evaluation metric value of the *i*-th sample, *n* is the number of samples, and *m* is the number of evaluation metrics.

Step 2: Standardize the sample of (*i*) evaluation indicators and (*j*) indicators.

(10) $$x_{i,j}=\frac{x_{i,\dot{J}}-\min\left(x_{1,j}\cdots x_{i,j}\right)}{\max\left(x_{1,j},\cdots ,x_{i,j}\right)-\min\left(x_{1,j},\cdots ,x_{i,j}\right)}$$

Step 3: Measure the sample ratio and calculate the sample mean, where the indicator serves as a large-scale framework and the sample size serves as the cyclie *x*. Calculate the proportion $F_{ij}$ of the *i*th sample value under the *j*th assessment indicator to the assessment indicator.

(11) $$F_{ij}=\frac{\sum_{i=1}^{n}x_{i,j}}{n}$$

Step 4: Calculate the sample mean, where the indicator serves as a large scale framework and the number of samples serves as the *x* of the loop, so it remains the same type as above.

(12) $$\rho_{j}=\frac{\sum_{i=1}^{n}x_{i,j}}{n}$$

Step 5: Calculate the sample standard deviation

(13) $$s_{j}=\sqrt{\frac{\sum_{i=1}^{n}{\left(x_{i}-\rho_{j}\right)}^{2}}{n-1}}$$

Step 6: Calculate the coefficient of variation CV

(14) $$CV=\frac{s_{j}}{\rho_{j}}$$

Step 7: Calculate the weights $\omega_{j}$

(15) $$\omega_{j}=\frac{CV_{j}}{\sum_{j=1}^{m}CV_{j}}$$

Step 8: Calculate the comprehensive score

(16) $$s_{i}=\Sigma_{j=1}^{m}\omega_{j}F_{i,j}$$

#### 3.2.2. Evaluation Score

The evaluation level of industrial internet network security is determined according to the indicator framework table established in Section 3.2.1. By comparing the collected data, the evaluation indicators are divided into five evaluation levels: very low, low, medium, high, and very high. The evaluation interval of quantitative data is determined on the basis of actual surveys. Compared with complex systems, the use of semi-quantitative information reasoning methods to handle safety indicators has significant advantages.

The fourth layer of the model is designed to address external threats to software and hardware devices, considering both the frequency and severity of the attacks. Therefore, all attack frequencies can be represented by Equation (17), and all attack severities can be represented by Equation (18).

(17) $$r_{xyz1},x=1,2;y=1,2,3;z=1,2,3,4,5$$

(18) $$r_{xyz2},x=1,2;y=1,2,3;z=1,2,3,4,5$$

The reference levels for the design of the different evaluation indicators mentioned above are shown in Table 2.

### 3.3. Establish a Data Fusion Model for the Four Level Evaluation Indicators

**Figure 3:**
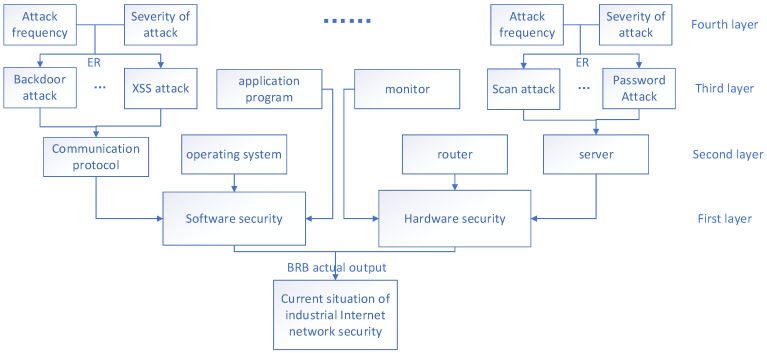
Fusion process of indicators at all levels.

**Figure 4:**
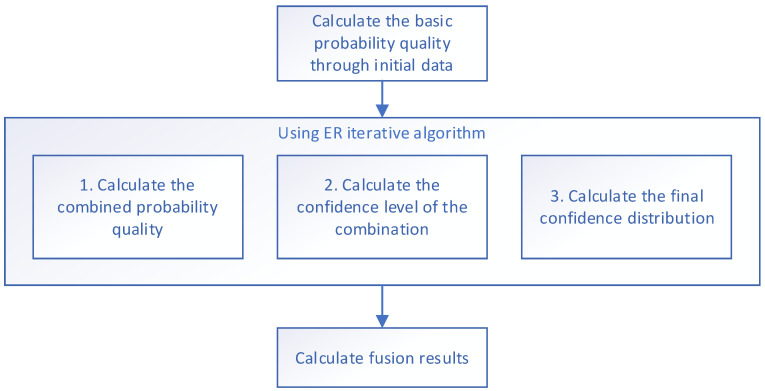
The ER iterative algorithm used.

By progressively integrating evaluation indicators at each level, uncertainties are systematically amalgamated. The resulting fused outcome serves as the definitive output for the final evaluation of the model. The fusion process is shown in Figure 3 and Figure 4, which show the ER iterative algorithm used. This ultimate output undergoes assessment using the initially fused indicators, providing a detailed evaluation of the model’s specific capabilities in the industrial internet environment.

Step 1: Establish the initial confidence level for each assessment tier, with the evaluation index denoted as $r_{i}$ for each tier. In this representation, *i* represents the rule, and n signifies the confidence level associated with the output segment. Θ represents the global ignorance.

(19) $$r_{i}=\left\{\left(D_{n},\beta_{n,j}\right),\left(\Theta ,\beta_{\Theta ,j}\right);i=1,\ldots ,L;n=1,\ldots ,N\right\}$$

Step 2: Calculating the Basic Probability Mass

Based on confidence level $\rho_{n,i}$ the basic probability mass of the evaluation index $r_{i}$ is calculated:

(20) $$m_{n,i}=\omega_{i}\beta_{n,i}$$

(21) $$m_{\theta ,i}=1-\omega_{i}\sum_{n=1}^{N}\rho_{n,i}$$

(22) $${\bar{m}}_{\Theta ,i}=1-\omega_{i}$$

(23) $${\tilde{m}}_{\theta ,i}=\omega_{i}\left(1-\sum_{n=1}^{N}\beta_{n,i}\right)$$

Step 3: Fusion Algorithm

Using the principle of the Dempster rule, multi-level indicators are graded and integrated. The attack frequency and severity of the four evaluation indicators are integrated in the framework table of the four evaluation indicators.${\;m}_{n,i}$ represents the basic probability mass of the nth evaluation level in the *i*th evaluation criterion.

(1) Calculate the probability mass of the combination

(24) $$m_{n,r\left(2\right)}=K_{0}\left[m_{n,1}m_{n,2}+m_{n,1}m_{\theta ,2}+m_{\theta ,1}m_{n,2}\right]$$

(25) $$m_{\theta ,1}={\bar{m}}_{\theta ,1}+{\tilde{m}}_{\theta ,1}$$

(26) $${\tilde{m}}_{\theta ,r\left(2\right)}=K_{0}\left[{\tilde{m}}_{\theta ,1}m_{\theta ,2}+{\tilde{m}}_{\theta ,1}{\bar{m}}_{\theta ,2}+{\bar{m}}_{\theta ,1}{\tilde{m}}_{\theta ,2}\right]$$

(27) $${\bar{m}}_{\theta ,r\left(2\right)}=K_{0}{\bar{m}}_{\theta ,1}{\bar{m}}_{\theta ,2}$$

(28) $$K_{0}={\left[1-\sum_{i=1}^{N}\sum_{j=1i\neq j}^{N}m_{i,1}m_{j,2}\right]}^{-1}$$

(2) Calculate the combined confidence level. $\beta_{\Theta ,i}$ refers to the unassigned confidence of the ith evaluation index. $\beta_{n,i}$ refers to the confidence assigned to the nth evaluation level in the ith evaluation index.
  (29) $$r\left(2\right)=\left\{\left(D_{n},\beta_{n,r\left(2\right)}\right),\left(\Theta ,\beta_{\Theta ,r\left(2\right)}\right),n=1,\ldots ,N\right\}$$
  (30) $$\beta_{n,r\left(2\right)}=\frac{m_{n,r\left(2\right)}}{1-m_{\Theta ,r\left(2\right)}}$$
  (31) $$\beta_{ta,r\left(2\right)}=\frac{m_{\Theta ,r\left(2\right)}}{1-m_{\Theta ,r\left(2\right)}}$$

(3) Calculate the final confidence distribution.

According to the fusion process of indicators, the final confidence distribution of the evaluation level is obtained using cyclic Equations (23)–(30). $m_{\Theta ,i}$ represents the unassigned basic probability mass in the ith evaluation indicator. $u\left(D_{n}\right)$ represents the utility set of the evaluation level $D_{n}$.

(32) $$r\left(L\right)=\left\{\left(\theta_{n},\beta_{n,r\left(L\right)}\right),\left(\Theta ,\beta_{\Theta ,r\left(L\right)}\right),n=1,\cdots ,N\right\}$$

Step 4: The fusion result of the model is

(33) $$u=\sum_{n=1}^{N}u\left(D_{n}\right)\beta_{n,r\left(L\right)}$$

The fusion result $u\left(D_{n}\right)$ of the model is obtained through Equation (32), so that the value range of the data is mapped to between [0, 1], and the security index of the industrial internet of the final fusion result is negatively correlated. The higher the result is, the greater the potential threat is; otherwise, the more secure it is.

### 3.4. The Evaluation Model of BRB-s Based on S-CMA-ES

Considering that the selective constraints of the model on experts will abandon some data, insufficient data will lead to a decrease in modeling accuracy. Therefore, we can try to construct a single choice strategy between different subclasses, which is in line with the idea of the S-CMA-ES algorithm.

Use the software security module and hardware security module integrated into the first layer as inputs to evaluate the overall network security score of the model. From this, the model can be described as:

(34) $$\left\{\begin{array}{l} R_{k}:\;\;If\;SW\;is\;x_{1}^{k}\wedge HW\;is\;x_{2}^{k}\\ Then\;y\;is\;\left\{\left(D_{1},\beta_{1,k}\right),\ldots ,\left(D_{N},\beta_{N,k}\right)\right\}\\ With\;rule\;weight\;\theta_{k}\;and\;attribute\;weight\;\delta_{1},\delta_{2} \end{array}\right.$$

**Figure 5:**
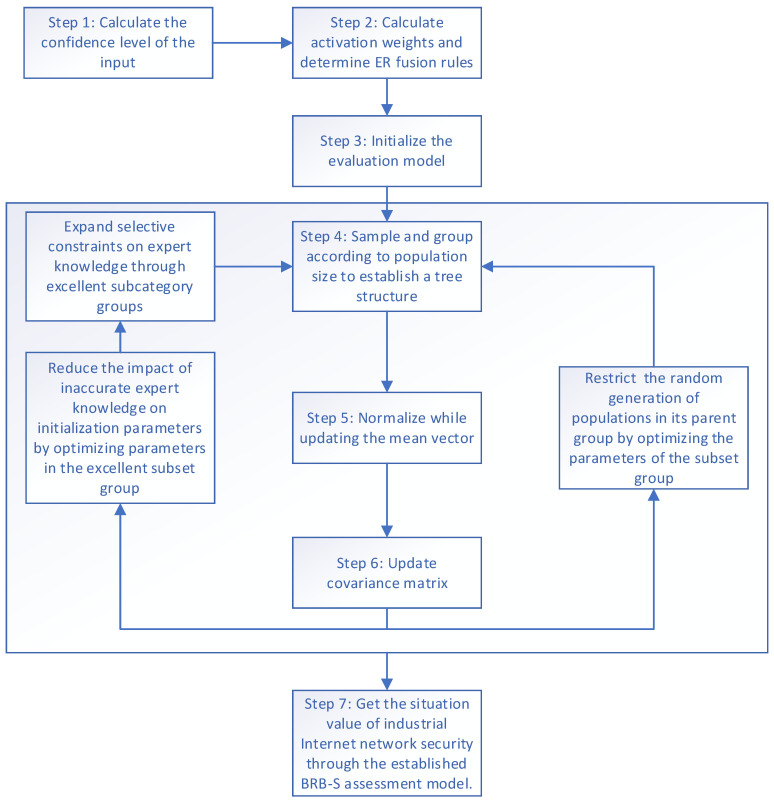
Process of BRB-s model.

The range of *k* is from 1 to *K*, representing the *k*th rule in the model. For the *k*th rule, the two input attributes are $x_{1}^{k}$ and $x_{2}^{k}$, followed by $\beta_{n,k}$, where *k* represents the confidence level related to the nth evaluation level of the *k*-th rule, *θk* represents the rule weight assigned to the kth rule, and $\delta_{1}$ and $\delta_{2}$ are the weights of the software security module and hardware security module for the input attributes in the model. *θk* represents the weight assigned to the *k*-th criterion. In addition, $\delta_{1},$ and $\delta_{2},$ correspond to the weights assigned to the two input attributes. This model takes the established four-level evaluation indicators as evaluation parameters and combines with the S-CMA-ES algorithm to build a BRB-s evaluation model for industrial internet network security with selective constraints on expert knowledge. The process of the BRB-s model is shown in Figure 5.

Step 1: Calculate the confidence level of the input.

The input data are imported through Equation (34); the confidence levels corresponding to the input attributes are calculated; and the input prerequisite attributes are converted to a unified format.

(35) $$\alpha_{j}^{i}=\left\{\begin{array}{l} \begin{array}{l} \frac{v_{i\left(k+1\right)}-v_{i}^{*}}{v_{i\left(k+1\right)}-v_{ik}}\;j=k,v_{ik}\leq v_{i}^{*}\leq v_{i\left(k+1\right)}\\ \frac{v_{i}^{*}-v_{ik}}{v_{i\left(k+1\right)}-v_{ik}}j=k+1\\ 0\;j=1\ldots L\;,\;j\neq k\;and\;k+1 \end{array} \end{array}\right.$$

The total matching of all attributes is calculated using Equation (35) based on the attribute matching degree obtained from Equation (34). $\alpha_{i}^{j}$ represents the matching degree of the *i*th attribute in the *j*th confidence rule.${\;w}_{k}$ represents the activation weight of the *k*-th belief rule.

(36) $$\alpha_{k}=\prod_{i=1}^{n}{\left(\alpha_{k}^{i}\right)}^{\delta_{i}}$$

Step 2: Calculate activation weights and determine ER fusion rules.

(37) $$w_{k}=\frac{\theta_{k}\alpha_{k}}{\sum_{i=1}^{K}\theta_{i}\alpha_{k}}0\leq w_{k}\leq 1,\sum_{k=1}^{K}w_{k}=1$$

After activating the belief rules, the ER parsing algorithm is used to combine the rules, and the calculation formula is:

(38) $$\beta_{n}=\frac{\mu \left[\begin{array}{c} \prod_{k=1}^{L^{\prime }}\left(w_{k}\beta_{n,k}+1-w_{k}\sum_{j=1}^{N}\beta_{j,k}\right)-\\ \prod_{k=1}^{L^{\prime }}\left(1-w_{k}\sum_{j=1}^{N}\beta_{j,k}\right) \end{array}\right]}{1-\mu \left[\prod_{k=1}^{L^{\prime }}\left(1-w_{k}\right)\right]}$$

(39) $$\mu =\left[\begin{array}{c} \sum_{n=1}^{N}\prod_{k=1}^{L^{\prime }}\left(w_{k}\beta_{1,k}+1-w_{k}\sum_{j=1}^{N}\beta_{j,k}\right)-\\ \left(N-1\right)\prod_{k=1}^{L^{\prime }}\left(1-w_{k}\sum_{j=1}^{N}\beta_{j,k}\right) \end{array}\right]$$

Step 3: Initialize the evaluation model.

The constraint process of the evaluation of the industrial internet in the BRB-s model is as follows. The application of the S-CMA-ES algorithm promotes the optimization of the program process, so that the optimized path and model can be selected more accurately and reasonably on the optimized road.

(40) $$\min MSE\left(\theta_{k},\beta_{n,k},\delta_{\dot{I}}\right)$$

(41) $$S\cdot t\cdot \sum_{n=1}^{N}\beta_{n,k}=1,k=1,\cdots K$$

(42) $$0\leq \beta_{n,k}\leq 1,0\leq \theta_{k}\leq 1$$

(43) $$0\leq \delta_{i}\leq 1,i=1,2$$

(44) $$MSE\left(\theta_{k},\beta_{n,k},\delta_{i}\right)=\frac{1}{T}\sum_{\dot{i}=1}^{T}({output}_{estimated}-{output}_{actual}{)}^{2}$$

The minimum value obtained through Equation (39) represents the degree of optimization. In Equation (44), ${output}_{actual}$ represents the real state of the system, derived through the integration of four tiers of assessment metrics grounded in the current conditions, and which serves as the actual value to measure the optimization effect. ${output}_{estimated}$ represents the estimated output of the evaluation model, represented by ${output}_{estimated}=\sum_{n=1}^{N}u\left(D_{n}\right)\beta_{n}$. Moreover, *T* is used as the number of training samples. The goal is to reduce the *MSE* through optimization and improve the accuracy of the model evaluation.

(45) $$\Omega^{0}=\left\{{\rho_{1,1}\ldots \rho_{N,k},\theta }_{1}\ldots \theta_{k},\delta_{1}\cdots \delta_{M}\right\}$$

Step 4: Sampling and grouping

When obtaining the number of offspring, lambda represents the number of offspring.

(46) $$lambda=10+\left\lfloor 3\log\left(N\times k+k+M\right)\right\rfloor$$

The number of groups was obtained through subalgebras, and after grouping, the subalgebras of each group were obtained. Gra represents the number of offspring after grouping.

(47) $$Gra=\left\lceil 2\times \frac{lambda}{\left\lceil \left.\log_{4}(Iambda\right)\right\rceil }\right\rceil$$

Sampling was performed via the following formula. $pdm^{g}$ represents the average distribution of the population in the *g*-th generation.

(48) $$\Omega_{k}^{g+1}\sim pdm^{g}+s^{g}N\left(0,CM^{g}\right)k=1\ldots \lambda$$

(49) $$pdm^{g}+s^{g}N\left(0,CM^{g}\right)\sim N\left(pdm^{g},{\left(s^{g}\right)}^{2}CM^{g}\right)$$

Step 5: Normalize while updating the mean vector.

Standardize every equation constraint and confine the potential solutions within the feasible domain to satisfy the constraint conditions. Here, *kn* and *vn* denote the number of rules and the present number of reference values, respectively. $vn_{e}$ represents the number of variables involved in the equality constraints of the solution. kn represents the number of equality constraints in the solution.

(50) $$\Omega_{k}^{g+1}\left(1+vn_{e}\times \left(kn-1\right):vn_{e}\times kn\right)=\frac{\Omega_{k}^{g+1}\left(1+vn_{e}\times \left(kn-1\right):vn_{e}\times kn\right)}{\sqrt{\sum_{i=1}^{vn}{\Omega_{k}^{g+1}\left(i+vn_{e}\times \left(kn-1\right)\right)}^{2}}}$$

Normalization can be defined as:

(51) $$\Omega_{k}^{g+1}\left(1+vn_{e}\times \left(xn-1\right):vn_{e}\times xn\right)=1$$

A selection operation is performed using the following formula to update the average value and obtain the current mean vector. $\Omega_{k:\lambda }^{g+1}$ represents the *k*-th solution selected from the λ individuals in generation *g* + 1.

(52) $$\rho dm^{g+1}=\sum_{k=1}^{\tau }h_{k}\Omega_{k:\lambda }^{g+1}$$

Step 6: Revise the matrix representing a standard distribution.

When all constraints are satisfied within the population, the normal distribution matrix is updated according to Equation (53),and the evolutionary trend and search range of the population are updated. $h_{k}$ represents the weight of the *k*th solution. $ep^{g}$ represents the evolutionary path of the covariance matrix in the *g*th generation.

(53) $$CM^{g+1}=\left(1-a_{1}-a_{\tau }\right)CM^{g}+a_{1}ep^{g+1}{\left({ep}^{g+1}\right)}^{T}+a_{i}\sum_{k=1}^{\tau }h_{k}\left(\frac{\omega_{k:\lambda }^{g+1}-pdm^{g}}{s^{g}}\right){\left(\frac{\omega_{k:\lambda }^{g+1}-pdm^{g}}{s^{g}}\right)}^{T}$$

Then continue the update process: $a_{ep}$ represents the parameters of the evolutionary path.

(54) $$ep^{g+1}=\left(1-a_{ep}\right)ep^{g}+\sqrt{a_{ep}\left(2-a_{ep}\right){\left(\sum_{k=1}^{\tau }h_{k}^{2}\right)}^{-1}}\frac{{pdm}^{g+1}-{pdm}^{g}}{s^{g}}$$

Next is the update rule for the step size:

(55) $$s^{g+1}=s^{g}\exp\left(\frac{a_{s}}{d_{s}}\left(\frac{\left\|ep_{s}^{g+1}\right\|}{E\left\|N\left(0,I\right)\right\|}-1\right)\right)$$

Additionally, $p_{\sigma }$ is updated according to the following formula:

(56) $$ep_{s}^{g+1}=\left(1-a_{s}\right)ep_{s}^{g}+\sqrt{a_{s}\left(2-a_{ep}\right){\left(\sum_{k=1}^{\tau }h_{k}^{2}\right)}^{-1}}\times CM^{g-\frac{1}{2}}\frac{{pdm}^{g+1}-{pdm}^{g}}{s^{g}}$$

Repeat the above steps to calculate the minimum mean square error value of the group, retain the step size of the minimum mean square error value, record the step size, mean vector, and individual weight of the optimal subset at this time, determine the initial parameter size determined by expert knowledge based on the evaluation level of the rules of the optimal subset, and update the population range of the next group, as shown in Equations (57) and (58). ${ub}_{n,k}^{b}$ and ${ub}_{n,k}^{b}$ represent the upper and lower limits of the *k*th rule and nth evaluation index of the population in the *b*-th subset, respectively. ${xmean}_{n,k}^{b}$ represents the mean vector of the kth rule and nth evaluation index of the population in the *b*-th subset.

(57) $${ub}_{n,k}^{b+1}={ub}_{n,k}^{b}-\frac{1}{4\left({ub}_{n,k}^{b}-{xmean}_{n,k}^{b}\right)}$$

(58) $${lb}_{n,k}^{b+1}={lb}_{n,k}^{b}-\frac{1}{4\left({lb}_{n,k}^{b}-{xmean}_{n,k}^{b}\right)}$$

Step 7: Utility calculation:

Finally, the situation value of industrial internet network security is obtained through the established BRB-s model.

(59) $$y=\sum_{n=1}^{N}u\left(D_{n}\right)\beta_{n}$$

Due to the challenges of data collection in the industrial internet environment, a lack of sufficient data has become the norm. In situations of data insufficiency, because different populations initially use different optimization strategies, selective constraints on expert knowledge can be expanded by utilizing the excellent subcategory group. When data are insufficient, the data within a single subcategory group become even more limited. Therefore, the optimization parameters from the excellent subcategory group will impose more significant restrictions on its parent cluster. This means that during the entire iterative process, when updating the normal distribution matrix, it is less likely to fall into a local optimum. Additionally, the expert knowledge system obtained through selective constraints on expert knowledge can enhance the model’s decision-making ability. As a result, when facing data insufficiency, the impact from this approach will be much smaller compared to other evaluation models.

## 4. Case Studies

To validate the accuracy of the proposed model in industrial internet evaluations, this study utilized the TON_IoT dataset provided by the University of New South Wales, Canberra campus [29,30,31]. This dataset was collected from a real-world, large-scale network designed within the University’s network infrastructure and the Canberra IoT Testbed, creating a novel testing platform for Industry 4.0 networks. The platform was deployed using multiple virtual machines and hosts, and various attack techniques were applied to network devices. The data used in this experiment specifically refer to the files in the “Train_Test_dataset” folder. Due to the dataset’s general applicability to industrial IoT scenarios, the constructed experimental platform also demonstrates broad relevance to industrial IoT environments.

### 4.1. Formulation of Problems

**Figure 6:**
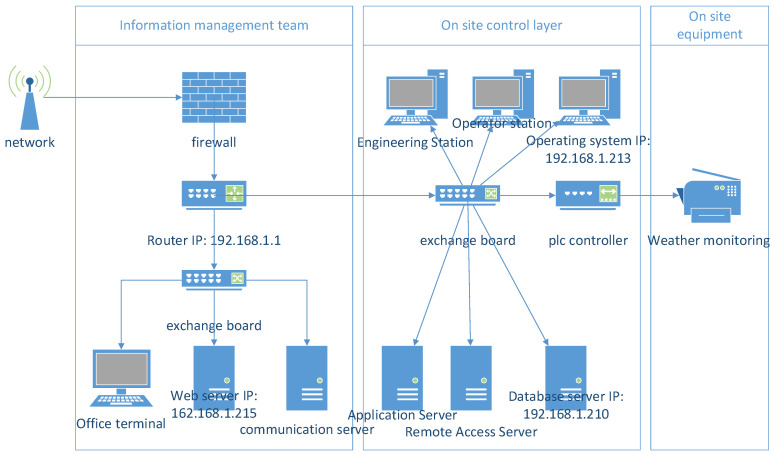
Experimental structure diagram.

Based on the dataset, test platform environment, and device descriptions mentioned in the literature, an experimental topology was constructed, as shown in Figure 6. This dataset includes attack data targeting multiple devices. The software devices selected in this study are Modbus (9000-K V9.1.1), a device with the IP address 192.168.1.213 (Kai), and a web application device with the IP address 192.168.1.215. The selected hardware devices include a server with the IP address 192.168.1.210 and a router with the IP address 192.168.1.1.

In the simulated experiment, the main types of attacks included scan attacks, injection attacks, XXS attacks, as well as ransomware attacks. Each device in the experiment was subjected to 4–5 major types of attacks. The attack data for each day were divided into 96 groups by time intervals, with each group representing a 15-min period. The frequency of each type of attack within these 96 groups was determined based on the number of occurrences of different attack types during the corresponding time intervals.

### 4.2. Gradual Fusion Experiment

**Figure 7:**
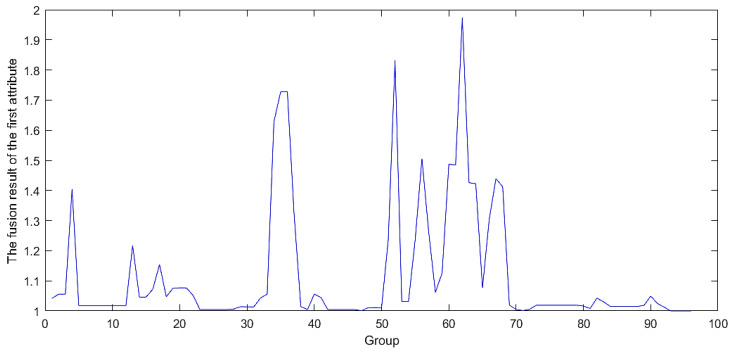
Fusion results of hardware security.

**Figure 8:**
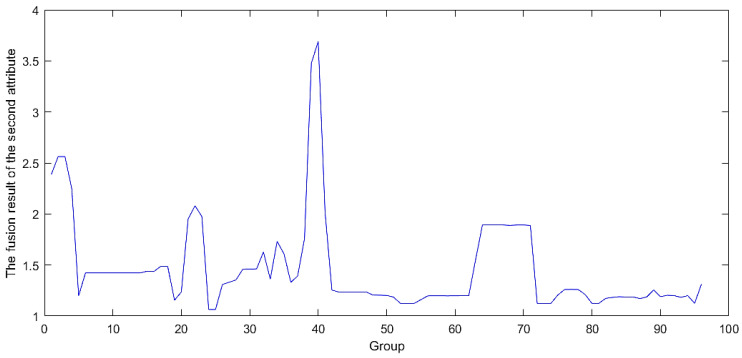
Fusion results of software security.

**Figure 9:**
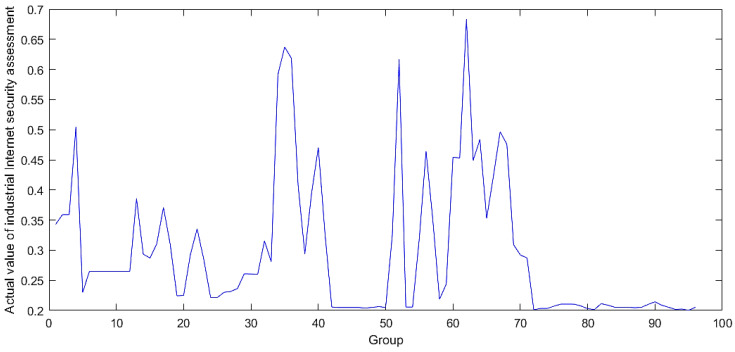
Actual results of the final action.

By sequentially fusing the four-level evaluation metrics mentioned in Section 3.2 and Section 3.3, the issue of fusing attack frequency and attack severity between different attack types was transformed into a problem focused solely on the software security module and the hardware security module. The attack frequency and severity data obtained from the 96 groups were fused in sequence. The progressively obtained software security and hardware security metrics were used as the first and second antecedents in the BRB-s model, as shown in Figure 7 and Figure 8. The final fused data between these two were used as the actual output of the BRB-s model, as illustrated in Figure 9.

The data after fusion shown in Figure 9 indicate that the initial four clusters exhibited high levels of security, suggesting that no severe damage would occur during an attack. However, starting from the fifth group, the security posture value rose sharply, indicating a significant attack at that point. Between groups 5 and 35, the security returned to normal, but another notable attack occurred in group 36. Finally, there was another surge between groups 51 and 71.

### 4.3. Establishment of an Industrial Internet Network Security Situation Assessment Model Based on ER and BRB-s

After sequentially merging the four assessment indicators using the ER fusion strategy, two evaluation metrics were obtained: hardware security and software security. These metrics are defined with five assessment levels: Excellent (*A*), Good (*B*), Average (*C*), Poor (*D*), and Severe (*E*), with the specific descriptions as follows:

(60) $$y=\left\{D_{1},D_{2},D_{3},D_{4},D_{5}\right\}=\left\{A,B,C,D,E\right\}$$

The following industrial internet assessment model is obtained through the specified assessment strategy guidelines.

(61) $$\left\{\begin{array}{l} R_{k}:If\;SW\;is\;x_{1}^{k}\;\wedge \;HW\;is\;x_{2}^{k},\;Then\;y\;is\\ \left\{\begin{array}{c} \left(A,\beta_{1,k}\right),\left(B,\beta_{2,k}\right),\left(C,\beta_{3,k}\right),\left(D,\beta_{4,k}\right),\left(E,\beta_{5,k}\right) \end{array}\right\}\\ With\;rule\;weight\;\theta_{k}\;and\;attribute\;weight\;\delta_{1}\;\delta_{2} \end{array}\right.$$

The evaluation results of the initial conditions and outcomes were classified into five levels: Excellent, Good, Average, Poor, and Dangerous. The reference values for the two input premise attributes were set to 1, 2, 3, 4, and 5, as shown in Table 3, while the reference values for the output were set to 0.2, 0.4, 0.6, 0.8, and 1, as shown in Table 4. These reference values represent intervals derived from expert knowledge, serving as the initial evaluation criteria for the model. Therefore, based on the five reference values set by expert knowledge and the two premise attributes, 25 initial confidence levels were determined, as shown in Table 5.

### 4.4. Testing and Training

**Figure 10:**
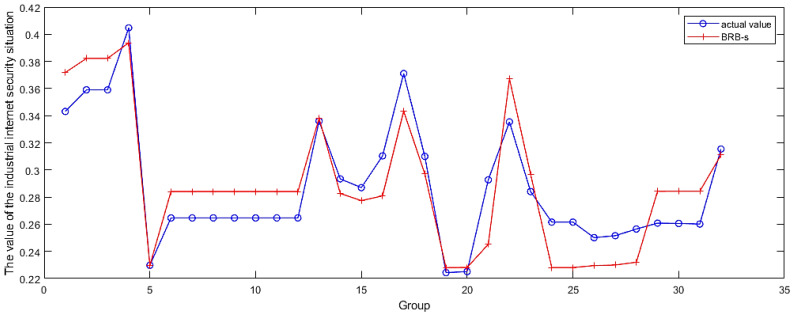
The evaluation effect of the BRB-s model.

Preprocessed data were trained and tested via the evaluation model established in Section 4.3. Sixty-four samples were randomly selected to adjust and optimize the initial parameters. By using the BRB-s model to constrain unreasonable expert knowledge throughout the entire training phase, the accuracy of evaluations in subsequent subclasses will continue to increase. Subsequently, based on the final evaluation results, the remaining 32 data points were subsequently evaluated and used as test data to assess the accuracy of the model. The optimized confidence levels are shown in Table 6. This study verified the effectiveness of the model through 10 rounds of experiments. The results are shown in Figure 10.

### 4.5. Comparison Analysis

**Figure 11:**
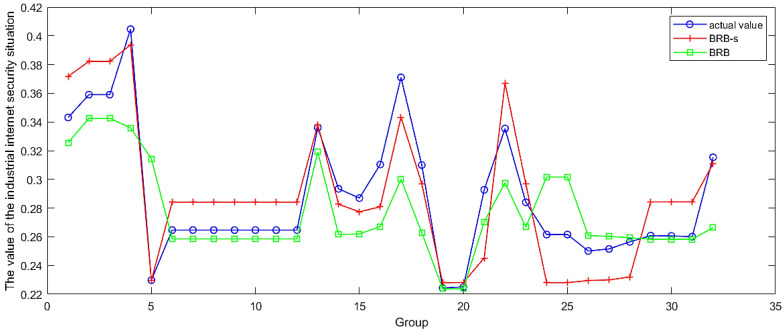
Comparison results between the BRB model and the BRB-s model.

**Figure 12:**
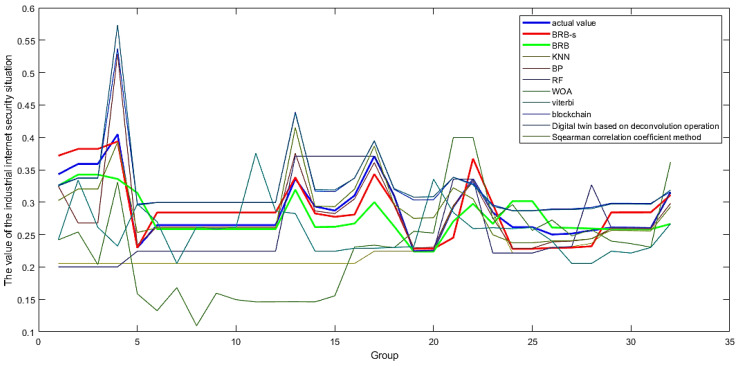
Comparison of various BRB models and other evaluation models.

First, a comparative analysis was conducted between the model optimized using BRB-s and the ordinary BRB optimization model. The main purpose of this study is to verify the problem of reduced evaluation accuracy caused by uncertain elements in expert knowledge when the BRB-s model is used. The final data obtained through BRB-s optimization are shown in Figure 10. In Figure 11, the BRB-s model was compared with the ordinary BRB model, and in Figure 12, the BRB model was compared with other traditional methods.

To prove the superiority of the BRB-s model, this model was compared with four common model evaluation methods, as well as the evaluation strategies of several commonly used methods in the past and a popular evolutionary strategy evaluation model. The K-Nearest Neighbors (KNN), Backpropagation Neural Network (BP), and Random Forest (RF) models were used as traditional evaluation models for comparison. One of the four common models was chosen, and the comparison results of the evaluation models established among them are shown in Figure 12.

Through 10 experiments, the evaluation results of each model were matched with the fused actual values, and SPSS statistical software (SPSS 27 Windows-x64) was used to obtain the average difference between the two groups and the actual values. The Cohen’s d test results obtained are shown in Table 7, and the average covariance values obtained are shown in Table 8.

By comparing the MSE line graph composed of the model evaluation results and the actual values in Figure 12, it can be proven that the BRB-s industrial internet network security evaluation model proposed in this paper is relatively accurate, effective, and reliable. By comparing the experimental results, the following conclusions can be drawn.

(1) In the comparison of the MSE sizes among the 10 models shown in Table 8, the average MSE values obtained by modeling with BRB-s and the feature selection method are 3.5 × $10^{-4}$ and 6.2 × $10^{-4}$, respectively, which have significant advantages over other evaluation models in such environments. This is because simple quantitative analysis can lead to inaccurate evaluation levels, so the BRB-s evaluation model that combines expert systems and quantitative data will be particularly advantageous in this situation.
(2) In Figure 11, the evaluation levels of the ordinary BRB model and the BRB-s model on the same set of data are compared, and the advantages of the BRB-s model over the BRB model can be intuitively felt. The BRB-s model selectively constrains inaccurate expert knowledge, effectively reducing the adverse effects of inaccurate expert knowledge on the evaluation itself. The experiment intuitively proved that adding selective constraints to expert knowledge can indeed improve the evaluation level of the model.
(3) By comparing the Cohen’s d value in Table 8, it can be concluded that the Cohen’s d value of the BRB-s model is less than 0.2. Compared with the actual situation, the evaluation system built using the BRB-s model has fewer differences and can accurately evaluate the security level of the entire industrial internet network. Similar evaluation models are also built using the feature selection method, and the model built using the spy selection method also has good evaluation results in this experimental environment. In addition, evaluation models constructed using other algorithms cannot produce good evaluation results and have significant differences from the BRB-s model.
(4) The differences between the BRB-s model and other evaluation models in Table 8 and Table 8 are comprehensively compared. Considering the special situation of the industrial internet environment, the model in this experimental environment first needs to be able to combine quantitative data and qualitative knowledge. Secondly, because the qualitative knowledge represented by expert knowledge is fuzzy and inaccurate to a certain extent, it also needs the ability to constrain inaccurate expert knowledge, which coincides with BRB-s. Therefore, using the BRB-s model in this environment will yield better evaluation results.

## 5. Conclusions

This paper presents an industrial internet situation assessment model, BRB-s, designed using the S-CMA-ES algorithm. The model extends the selective constraints on expert knowledge, optimizes the evolutionary path, and reduces the selection of unnecessary paths. At the same time, it provides an effective modeling strategy to address challenges posed by the complexity of industrial internet systems, such as insufficient data and poor data quality. Through nine comparative tests, the BRB-s model is further demonstrated to outperform other evaluation models in network security assessment, particularly in industrial internet environments with limited or low-quality data. Compared to other evaluation methods, this modeling strategy is better suited to complex industrial internet network environments. Based on the evaluation results, administrators can provide safe and reliable recommendations and take timely measures when security incidents are detected, thereby enhancing overall risk assessment capabilities. However, the model still faces challenges regarding the unreliability of expert knowledge. Specifically, it cannot guarantee the reliability of expert knowledge in situational awareness prediction. To address the issue of unreliable expert knowledge, the model’s evaluation accuracy methods will further be enhanced and in-depth research on situational awareness prediction for industrial internet network security will be conducted, aiming to improve the components of industrial internet security situational awareness prediction.

## Figures and Tables

**Table 1:** Industrial internet Network Security Situation Assessment Framework.

First Layer	Second Floor	Third Layer	Fourth Layer
Software security $\left(r_{1}\right)\left(\omega_{1}=0.3479\right)$	Communication protocol $\left(r_{11}\right)\left(\omega_{11}=0.5246\right)$	Backdoor attack $\left(r_{111}\right)\left(\omega_{111}=0.1462\right)$ Injection attack $\left(r_{112}\right)\left(\omega_{112}=0.2221\right)$ Password attack $\left(r_{113}\right)\left(\omega_{113}=0.1612\right)$ Attack aircraft $\left(r_{114}\right)\left(\omega_{114}=0.1583\right)$ XSS aircraft $\left(r_{115}\right)\left(\omega_{115}=0.3122\right)$	Attack frequency $\left(r_{1111}\right)\left(\omega_{1111}=0.5204\right)$ Severity of attack $\left(r_{1112}\right)\left(\omega_{1112}=0.4851\right)$ Attack frequency $\left(r_{1121}\right)\left(\omega_{1121}=0.5385\right)$ Severity of attack $\left(r_{1122}\right)\left(\omega_{1122}=0.4615\right)$ Attack frequency $\left(r_{1131}\right)\left(\omega_{1131}=0.5242\right)$ Severity of attack $\left(r_{1132}\right)\left(\omega_{1132}=0.4758\right)$ Attack frequency $\left(r_{1141}\right)\left(\omega_{1141}=0.5047\right)$ Severity of attack $\left(r_{1142}\right)\left(\omega_{1142}=0.4953\right)$ Attack frequency $\left(r_{1151}\right)\left(\omega_{1151}=0.5169\right)$ Severity of attack $\left(r_{1152}\right)\left(\omega_{1152}=0.4831\right)$
	Operating system $\left(r_{12}\right)\left(\omega_{12}=0.1218\right)$	Dos aircraft $\left(r_{121}\right)\left(\omega_{121}=0.1803\right)$ Password attack $\left(r_{122}\right)\left(\omega_{122}=0.3290\right)$ Ransomware $\left(r_{123}\right)\left(\omega_{123}=0.2414\right)$ Backdoor attack $\left(r_{124}\right)\left(\omega_{124}=0.2493\right)$	Attack frequency $\left(r_{1211}\right)\left(\omega_{1211}=0.4994\right)$ Severity of attack $\left(r_{1212}\right)\left(\omega_{1212}=0.5006\right)$ Attack frequency $\left(r_{1221}\right)\left(\omega_{1221}=0.5016\right)$ Severity of attack $\left(r_{1222}\right)\left(\omega_{1222}=0.4985\right)$ Attack frequency $\left(r_{1231}\right)\left(\omega_{1231}=0.5004\right)$ Severity of attack $\left(r_{1232}\right)\left(\omega_{1232}=0.4996\right)$ Attack frequency $\left(r_{1241}\right)\left(\omega_{1241}=0.500\right)$ Severity of attack $\left(r_{1242}\right)\left(\omega_{1242}=0.500\right)$
	Application program $\left(r_{13}\right)\left(\omega_{31}=0.3536\right)$	Injection attack $\left(r_{131}\right)\left(\omega_{131}=0.1980\right)$ Password attack $\left(r_{132}\right)\left(\omega_{132}=0.1626\right)$ XSS attack $\left(r_{133}\right)\left(\omega_{133}=0.2564\right)$ Scan estimation $\left(r_{134}\right)\left(\omega_{134}=0.3830\right)$	Attack frequency $\left(r_{1311}\right)\left(\omega_{1311}=0.5051\right)$ Severity of attack $\left(r_{1312}\right)\left(\omega_{1312}=0.4949\right)$ Attack frequency $\left(r_{1321}\right)\left(\omega_{1321}=0.5010\right)$ Severity of attack $\left(r_{1322}\right)\left(\omega_{1322}=0.4990\right)$ Attack frequency $\left(r_{1331}\right)\left(\omega_{1331}=0.5005\right)$ Severity of attack $\left(r_{1332}\right)\left(\omega_{1332}=0.4995\right)$ Attack frequency $\left(r_{1341}\right)\left(\omega_{1341}=0.5026\right)$ Severity of attack $\left(r_{1342}\right)\left(\omega_{1342}=0.4974\right)$
Hardware security $\left(r_{2}\right)\left(\omega_{2}=0.6521\right)$	Monitor $\left(r_{21}\right)\left(\omega_{21}=0.3464\right)$	Ddos attack $\left(r_{211}\right)\left(\omega_{211}=0.1749\right)$ Injection attack $\left(r_{212}\right)\left(\omega_{212}=0.2498\right)$ Password attack $\left(r_{213}\right)\left(\omega_{213}=0.1647\right)$ Backdoor attack $\left(r_{214}\right)\left(\omega_{214}=0.1576\right)$ Ransomware $\left(r_{215}\right)\left(\omega_{215}=0.2530\right)$	Attack frequency $\left(r_{2111}\right)\left(\omega_{2111}=0.5228\right)$ Severity of attack $\left(r_{2112}\right)\left(\omega_{2112}=0.4772\right)$ Attack frequency $\left(r_{2121}\right)\left(\omega_{2121}=0.5324\right)$ Severity of attack $\left(r_{2122}\right)\left(\omega_{2122}=0.4676\right)$ Attack frequency $\left(r_{2131}\right)\left(\omega_{2131}=0.5237\right)$ Severity of attack $\left(r_{2132}\right)\left(\omega_{2132}=0.4763\right)$ Attack frequency $\left(r_{2141}\right)\left(\omega_{2141}=0.5176\right)$ Severity of attack $\left(r_{2142}\right)\left(\omega_{2142}=0.4824\right)$ Attack frequency $\left(r_{2151}\right)\left(\omega_{2151}=0.5080\right)$ Severity of attack $\left(r_{2152}\right)\left(\omega_{2152}=0.4919\right)$
	Router $\left(r_{22}\right)\left(\omega_{22}=0.2589\right)$	Scan attack $\left(r_{221}\right)\left(\omega_{221}=0.2782\right)$ Ddos attack $\left(r_{222}\right)\left(\omega_{222}=0.0886\right)$ Dos attack $\left(r_{223}\right)\left(\omega_{223}=0.1808\right)$ Xss attack $\left(r_{224}\right)\left(\omega_{224}=0.1892\right)$ Injection attack $\left(r_{225}\right)\left(\omega_{225}=0.2634\right)$	Attack frequency $\left(r_{2211}\right)\left(\omega_{2211}=0.5041\right)$ Severity of attack $\left(r_{2212}\right)\left(\omega_{2212}=0.4959\right)$ Attack frequency $\left(r_{2221}\right)\left(\omega_{2221}=0.5017\right)$ Severity of attack $\left(r_{2222}\right)\left(\omega_{2222}=0.4983\right)$ Attack frequency $\left(r_{2231}\right)\left(\omega_{2231}=0.5151\right)$ Severity of attack $\left(r_{2232}\right)\left(\omega_{2232}=0.4849\right)$ Attack frequency $\left(r_{2241}\right)\left(\omega_{2241}=0.5057\right)$ Severity of attack $\left(r_{2242}\right)\left(\omega_{2242}=0.4943\right)$ Attack frequency $\left(r_{2241}\right)\left(\omega_{2241}=0.5195\right)$ Severity of attack $\left(r_{2242}\right)\left(\omega_{2242}=0.4805\right)$
	Server $\left(r_{23}\right)\left(\omega_{23}=0.3947\right)$	Scan attack $\left(r_{231}\right)\left(\omega_{231}=0.3127\right)$ Ddos attack $\left(r_{232}\right)\left(\omega_{232}=0.1607\right)$ Dos attack $\left(r_{233}\right)\left(\omega_{233}=0.1984\right)$ Injection attack $\left(r_{234}\right)\left(\omega_{234}=0.1878\right)$ Password attack $\left(r_{235}\right)\left(\omega_{235}=0.1404\right)$	Attack frequency $\left(r_{2311}\right)\left(\omega_{2311}=0.4993\right)$ Severity of attack $\left(r_{2312}\right)\left(\omega_{2312}=0.5007\right)$ Attack frequency $\left(r_{2321}\right)\left(\omega_{2321}=0.5157\right)$ Severity of attack $\left(r_{2322}\right)\left(\omega_{2322}=0.4843\right)$ Attack frequency $\left(r_{2331}\right)\left(\omega_{2331}=0.5081\right)$ Severity of attack $\left(r_{2332}\right)\left(\omega_{2332}=0.4919\right)$ Attack frequency $\left(r_{2341}\right)\left(\omega_{2341}=0.4995\right)$ Severity of attack $\left(r_{2342}\right)\left(\omega_{2342}=0.5005\right)$ Attack frequency $\left(r_{2241}\right)\left(\omega_{2241}=0.5076\right)$ Severity of attack $\left(r_{2242}\right)\left(\omega_{2242}=0.4924\right)$

**Table 2:** Reference values corresponding to the five evaluation levels.

Evaluating Indicator	VL	L	M	H	VH
$r_{xyz1}$	0 times/min	5 times/min	15 times/min	30 times/min	50 times/min
$r_{xyz2}$			Determined by expert knowledge		

**Table 3:** Outlines reference points and values pertaining to software and hardware.

Reference Point	A	B	C	D	E
reference value	1	2	3	4	5

**Table 4:** Reference values for the evaluation results.

Reference Point	A	B	C	D	E
reference value	0.2	0.4	0.6	0.8	1

**Table 5:** Optimizing confidence.

NO.	$\theta_{k}$	SW	HW	$\left\{\begin{array}{c} \left(A,\beta_{1,k}\right),\left(B,\beta_{2,k}\right),\left(C,\beta_{3,k}\right),\left(D,\beta_{4,k}\right),\left(E,\beta_{5,k}\right) \end{array}\right\}$
1	1	A	A	$\left\{\begin{array}{c} \left(A,1\right),\left(B,0\right),\left(C,0\right),\left(D,0\right),\left(E,0\right) \end{array}\right\}$
2	1	A	B	$\left\{\begin{array}{c} \left(A,0.5\right),\left(B,0.5\right),\left(C,0\right),\left(D,0\right),\left(E,0\right) \end{array}\right\}$
3	1	A	C	$\left\{\begin{array}{c} \left(A,0.5\right),\left(B,0.25\right),\left(C,0.25\right),\left(D,0\right),\left(E,0\right) \end{array}\right\}$
4	1	A	D	$\left\{\begin{array}{c} \left(A,0\right),\left(B,0.25\right),\left(C,0.25\right),\left(D,0.25\right),\left(E,0.25\right) \end{array}\right\}$
5	1	A	E	$\left\{\begin{array}{c} \left(A,2\right),\left(B,2\right),\left(C,2\right),\left(D,2\right),\left(E,2\right) \end{array}\right\}$
6	1	B	A	$\left\{\begin{array}{c} \left(A,0.5\right),\left(B,0.5\right),\left(C,0\right),\left(D,0\right),\left(E,0\right) \end{array}\right\}$
7	1	B	B	$\left\{\begin{array}{c} \left(A,0\right),\left(B,0.75\right),\left(C,0.25\right),\left(D,0\right),\left(E,0\right) \end{array}\right\}$
8	1	B	C	$\left\{\begin{array}{c} \left(A,0.2\right),\left(B,0.5\right),\left(C,0.1\right),\left(D,0.1\right),\left(E,0.1\right) \end{array}\right\}$
9	1	B	D	$\left\{\begin{array}{c} \left(A,0.1\right),\left(B,0.4\right),\left(C,0.2\right),\left(D,0.2\right),\left(E,0.1\right) \end{array}\right\}$
10	1	B	E	$\left\{\begin{array}{c} \left(A,0.1\right),\left(B,0.5\right),\left(C,0.2\right),\left(D,0\right),\left(E,0.2\right) \end{array}\right\}$
11	1	C	A	$\left\{\begin{array}{c} \left(A,0.2\right),\left(B,0.4\right),\left(C,0.3\right),\left(D,0\right),\left(E,0\right) \end{array}\right\}$
12	1	C	B	$\left\{\begin{array}{c} \left(A,0.1\right),\left(B,0.4\right),\left(C,0.4\right),\left(D,0.1\right),\left(E,0\right) \end{array}\right\}$
13	1	C	C	$\left\{\begin{array}{c} \left(A,0.05\right),\left(B,0.05\right),\left(C,0.5\right),\left(D,0.3\right),\left(E,0.1\right) \end{array}\right\}$
14	1	C	D	$\left\{\begin{array}{c} \left(A,0\right),\left(B,0\right),\left(C,0.4\right),\left(D,0.4\right),\left(E,0.2\right) \end{array}\right\}$
15	1	C	E	$\left\{\begin{array}{c} \left(A,0.1\right),\left(B,0.2\right),\left(C,0.5\right),\left(D,0.1\right),\left(E,0.1\right) \end{array}\right\}$
16	1	D	A	$\left\{\begin{array}{c} \left(A,0.1\right),\left(B,0.1\right),\left(C,0.1\right),\left(D,0.4\right),\left(E,0.3\right) \end{array}\right\}$
17	1	D	B	$\left\{\begin{array}{c} \left(A,0.1\right),\left(B,0.2\right),\left(C,0.1\right),\left(D,0.4\right),\left(E,0.2\right) \end{array}\right\}$
18	1	D	C	$\left\{\begin{array}{c} \left(A,0.1\right),\left(B,0.2\right),\left(C,0.1\right),\left(D,0.4\right),\left(E,0.2\right) \end{array}\right\}$
19	1	D	D	$\left\{\begin{array}{c} \left(A,0.1\right),\left(B,0.05\right),\left(C,0.15\right),\left(D,0.5\right),\left(E,0.2\right) \end{array}\right\}$
20	1	D	E	$\left\{\begin{array}{c} \left(A,0.1\right),\left(B,0.05\right),\left(C,0.1\right),\left(D,0.4\right),\left(E,0.35\right) \end{array}\right\}$
21	1	E	A	$\left\{\begin{array}{c} \left(A,0.15\right),\left(B,0.05\right),\left(C,0.2\right),\left(D,0.4\right),\left(E,0.2\right) \end{array}\right\}$
22	1	E	B	$\left\{\begin{array}{c} \left(A,0.05\right),\left(B,0.15\right),\left(C,0.2\right),\left(D,0.4\right),\left(E,0.2\right) \end{array}\right\}$
23	1	E	C	$\left\{\begin{array}{c} \left(A,0.05\right),\left(B,0.2\right),\left(C,0.2\right),\left(D,0.3\right),\left(E,0.25\right) \end{array}\right\}$
24	1	E	D	$\left\{\begin{array}{c} \left(A,0.1\right),\left(B,0.05\right),\left(C,0.05\right),\left(D,0.25\right),\left(E,0.55\right) \end{array}\right\}$
25	1	E	E	$\left\{\begin{array}{c} \left(A,0.05\right),\left(B,0.15\right),\left(C,0.2\right),\left(D,0.3\right),\left(E,0.3\right) \end{array}\right\}$

**Table 6:** Optimization confidence.

NO.	$\theta_{k}$	SW	HW	$\left\{\begin{array}{c} \left(E,\beta_{1,k}\right),\;\left(G,\beta_{2,k}\right),\;\left(C,\beta_{3,k}\right),\;\left(B,\beta_{4,k}\right),\;\left(D,\beta_{5,k}\right) \end{array}\right\}$
1	1	E	E	$\left\{\begin{array}{c} \left(E,0.452\right),\;\left(G,0.131\right),\;\left(C,0.162\right),\;\left(B,0.071\right),\;\left(D,0.184\right) \end{array}\right\}$
2	1	E	G	$\left\{\begin{array}{c} \left(E,0.339\right),\;\left(G,0.087\right),\;\left(C,0.088\right),\;\left(B,0.395\right),\;\left(D,0.091\right) \end{array}\right\}$
3	1	E	C	$\left\{\begin{array}{c} \left(E,0.201\right),\;\left(G,0.155\right),\;\left(C,0.033\right),\;\left(B,0.224\right),\;\left(D,0.387\right) \end{array}\right\}$
4	1	E	B	$\left\{\begin{array}{c} \left(E,0.443\right),\left(G,0.032\right),\left(C,0.059\right),\left(B,0.047\right),\left(D,0.419\right) \end{array}\right\}$
5	1	E	D	$\left\{\begin{array}{c} \left(E,0.043\right),\;\left(G,0.186\right),\;\left(C,0.162\right),\;\left(B,0.338\right),\;\left(D,0.271\right) \end{array}\right\}$
6	1	G	E	$\left\{\begin{array}{c} \left(E,0.047\right),\;\left(G,0.614\right),\;\left(C,0.278\right),\;\left(B,0.019\right),\;\left(D,0.042\right) \end{array}\right\}$
7	1	G	G	$\left\{\begin{array}{c} \left(E,0.158\right),\;\left(G,0.083\right),\;\left(C,0.171\right),\;\left(B,0.419\right),\;\left(D,0.170\right) \end{array}\right\}$
8	1	G	C	$\left\{\begin{array}{c} \left(E,0.161\right),\;\left(G,0.150\right),\;\left(C,0.462\right),\;\left(B,0.185\right),\;\left(D,0.042\right) \end{array}\right\}$
9	1	G	B	$\left\{\begin{array}{c} \left(E,0.201\right),\;\left(G,0.339\right),\;\left(C,0.113\right),\;\left(B,0.306\right),\;\left(D,0.041\right) \end{array}\right\}$
10	1	G	D	$\left\{\begin{array}{c} \left(E,0.123\right),\;\left(G,0.032\right),\;\left(C,0.100\right),\;\left(B,0.035\right),\;\left(D,0.710\right) \end{array}\right\}$
11	1	C	E	$\left\{\begin{array}{c} \left(E,0.024\right),\;\left(G,0.145\right),\;\left(C,0.054\right),\;\left(B,0.302\right),\;\left(D,0.475\right) \end{array}\right\}$
12	1	C	G	$\left\{\begin{array}{c} \left(E,0.062\right),\;\left(G,0.484\right),\;\left(C,0.308\right),\;\left(B,0.109\right),\;\left(D,0.037\right) \end{array}\right\}$
13	1	C	C	$\left\{\begin{array}{c} \left(E,0.278\right),\;\left(G,0.028\right),\;\left(C,0.167\right),\;\left(B,0.493\right),\;\left(D,0.034\right) \end{array}\right\}$
14	1	C	B	$\left\{\begin{array}{c} \left(E,0.346\right),\;\left(G,0.337\right),\;\left(C,0.029\right),\;\left(B,0.217\right),\;\left(D,0.071\right) \end{array}\right\}$
15	1	C	D	$\left\{\begin{array}{c} \left(E,0.022\right),\;\left(G,0.120\right),\;\left(C,0.285\right),\;\left(B,0.125\right),\;\left(D,0.448\right) \end{array}\right\}$
16	1	B	E	$\left\{\begin{array}{c} \left(E,0.857\right),\;\left(G,0.094\right),\;\left(C,0.020\right),\;\left(B,0.029\right),\;\left(D,0\right) \end{array}\right\}$
17	1	B	G	$\left\{\begin{array}{c} \left(E,0.068\right),\;\left(G,0.438\right),\;\left(C,0.248\right),\;\left(B,0.199\right),\;\left(D,0.047\right) \end{array}\right\}$
18	1	B	C	$\left\{\begin{array}{c} \left(E,0.045\right),\;\left(G,0.165\right),\;\left(C,0.032\right),\;\left(B,0.034\right),\;\left(D,0.723\right) \end{array}\right\}$
19	1	B	B	$\left\{\begin{array}{c} \left(E,0\right),\;\left(G,0.016\right),\;\left(C,0.058\right),\;\left(B,0.057\right),\;\left(D,0.869\right) \end{array}\right\}$
20	1	B	D	$\left\{\begin{array}{c} \left(E,0.004\right),\;\left(G,0\right),\;\left(C,0\right),\;\left(B,0.003\right),\;\left(D,0.993\right) \end{array}\right\}$
21	1	D	E	$\left\{\begin{array}{c} \left(E,0.526\right),\;\left(G,0.091\right),\;\left(C,0.194\right),\;\left(B,0.170\right),\;\left(D,0.019\right) \end{array}\right\}$
22	1	D	G	$\left\{\begin{array}{c} \left(E,0.066\right),\;\left(G,0.207\right),\;\left(C,0.153\right),\;\left(B,0.464\right),\;\left(D,0.110\right) \end{array}\right\}$
23	1	D	C	$\left\{\begin{array}{c} \left(E,0.309\right),\;\left(G,0.038\right),\;\left(C,0.133\right),\;\left(B,0.223\right),\;\left(D,0.297\right) \end{array}\right\}$
24	1	D	B	$\left\{\begin{array}{c} \left(E,0\right),\;\left(G,0.001\right),\;\left(C,0.006\right),\;\left(B,0.016\right),\;\left(D,0.977\right) \end{array}\right\}$
25	1	D	D	$\left\{\begin{array}{c} \left(E,0.414\right),\;\left(G,0.037\right),\;\left(C,0.076\right),\;\left(B,0.180\right),\;\left(D,0.293\right) \end{array}\right\}$

**Table 7:** Wilcoxon signed rank test results.

Model	P-CMA-ES	S-CMA-ES	KNN	BP	RF
Cohen’s d	0.2714	0.017693	0.8432	0.34791	0.548
Model	Whale optimization	intrusion detection	blockchain	Digital twin	Feature Selection
Cohen’s d	0.4287	0.3329	0.4163	0.3125	0.026397

**Table 8:** Average MSE values for different models.

Model	P-CMA-ES	S-CMA-ES	KNN	BP	RF
MSE	0.0011	0.00035	0.00342	0.00124	0.0051
Model	Whale optimization	intrusion detection	blockchain	Digital twin	Feature Selection
MSE	0.009122	0.00454	0.00229	0.00242	0.00062

## Data Availability

The data presented in this study are available on request from the corresponding author.
